# Supplementary material for: Non-apoptotic caspase-8 is critical for orchestrating exaggerated inflammation during severe SARS-CoV-2 infection
Source: Nat Commun. 2025 Nov 13;16:9822. doi: 10.1038/s41467-025-65098-z (PMC12615602; doi:10.1038/s41467-025-65098-z)
Supplement: Supplementary file 1 — Supplementary Information [file 41467_2025_65098_MOESM1_ESM.pdf]

Supplementary File

Non-apoptotic caspase-8 is critical for orchestrating exaggerated inflammation during severe SARS-CoV-2 infection.

Stefanie M. Bader[1,2]*, Lena Scherer[1,2], Reet Bhandari[1,2], Allan J. Motyer[1,2], James P. Cooney[1,2], Liana Mackiewicz[1], Merle Dayton[1], Dylan Sheerin[1,2], David V. L. Romero[1,2], Jan Schaefer[1,2], Jiyi Pang[1,2], Siqi Chen[1,3], Kael Schoffer[1], Le Wang[1,2], Xinyi Jin[1,2], Daniel Batey[1], Raymond K.H. Yip [1,2], Ishrat Zaman[1], Pradeep Rajasekhar[1,2], Matthew J. Gartner[4], Stephen Wilcox[1,2], Lachlan Whitehead[1,2], Smitha Rose Georgy[5], Ana Maluenda[1], Kathryn C. Davidson[1,2], Cody C. Allison[1], Rory Bowden[1,2], Kerstin Brinkmann[1,2], Marie-Liesse Asselin-Labat[1,2], Belinda Phipson[1,2], Maria C. Tanzer[1,2], Marco J. Herold[1,2,6,7], Andre L. Samson[1,2], James E. Vince[1,2], Andreas Strasser[1,2], Marc Pellegrini[1,2,8]*, Marcel Doerflinger[1,2]*

Affiliations

[1] The Walter and Eliza Hall Institute of Medical Research, Melbourne, Victoria, Australia.

[2] Department of Medical Biology, University of Melbourne, Melbourne, Australia.

[3] College of Life Sciences, Nankai University, Tianjin, China

[4] Department of Microbiology and Immunology, the University of Melbourne at the Peter Doherty Institute for Infection and Immunity, Melbourne, VIC 3000, Australia

[5] Section of Anatomic Pathology, Melbourne Veterinary School, Faculty of Science, University of Melbourne, Werribee, Victoria, Australia

[6] Olivia Newton-John Cancer Research Institute, Heidelberg, Victoria, Australia

[7] School of Cancer Medicine, La Trobe University, Heidelberg, Victoria, Australia

[8] Centenary Institute and University of Technology Sydney, Faculty of Science, School of Life Sciences, Sydney, NSW, Australia.

* Corresponding authors. Marcel Doerflinger, doerflinger.m@wehi.edu.au; Marc Pellegrini, m.pellegrini@centenary.org.au; Stefanie M. Bader, bader.s@wehi.edu.au

Figure S1

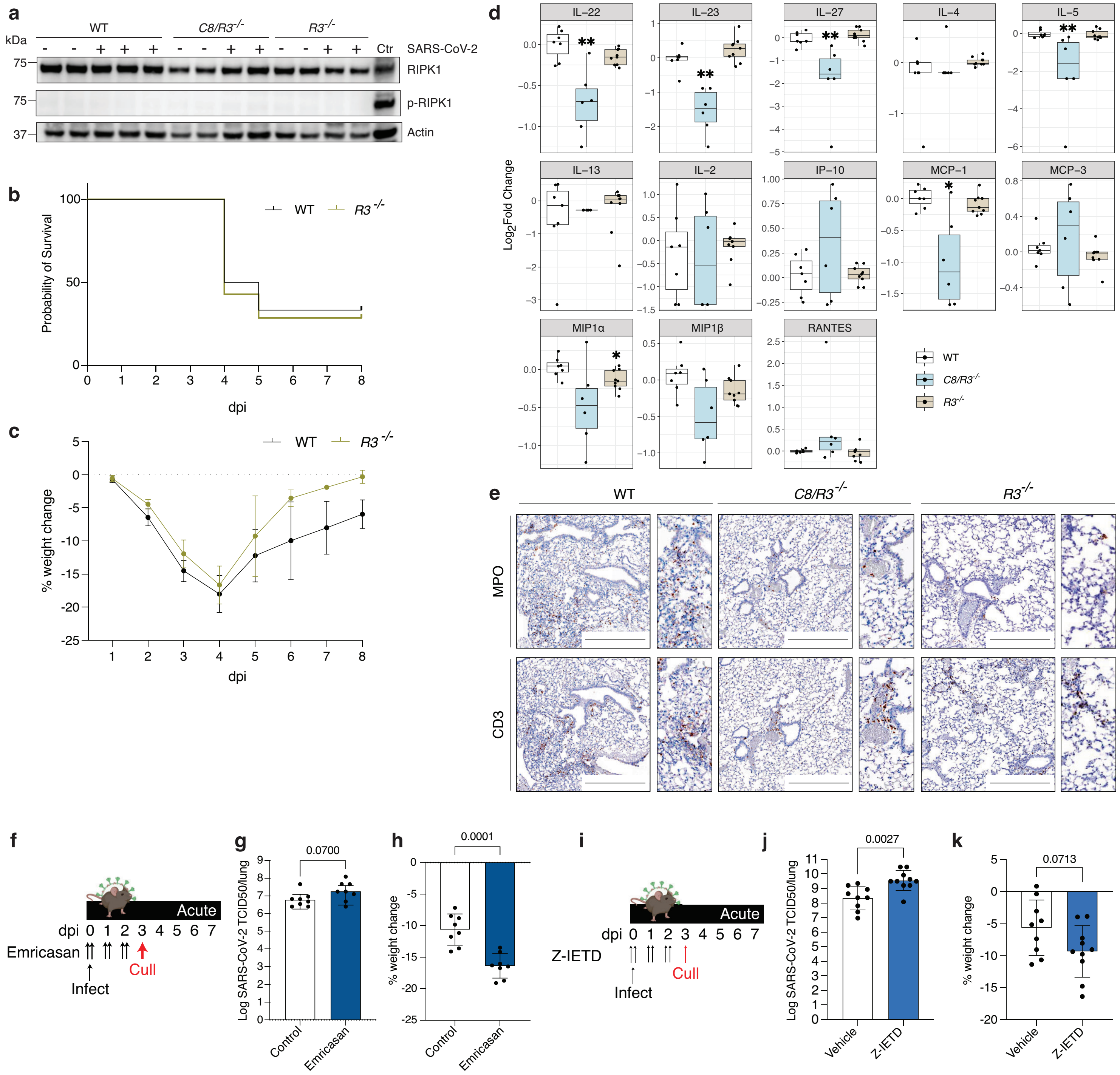

a
WT
C8/R3-/-
R3-/-
kDa
SARS-CoV-2
Ctr
RIPK1
p-RIPK1
Actin
b
Probability of Survival
dpi
WT
R3 -/-
c
% weight change
dpi
d
Log2Fold Change
IL-22
IL-23
IL-27
IL-4
IL-5
IL-13
IL-2
IP-10
MCP-1
MCP-3
MIP1α
MIP1β
RANTES
e
MPO
CD3
f
Acute
Emricasan
Infect
Cull
g
Log SARS-CoV-2 TCID50/lung
0.0700
Control
Emricasan
h
0.0001
i
Z-IETD
j
0.0027
Vehicle
k
0.0713

Fig. S1: Mice lacking caspase-8 have reduced levels of cytokines upon SARS-CoV-2 infection. (a) Western blot analysis of whole lungs from mock (-) or SARS-CoV-2 (10^4 TCID50) infected (+) WT, *C8/R3*$^{-/-}$ or *R3*$^{-/-}$ mice at 3 days after intranasal infection. Samples were probed for RIPK3 and phosphorylated RIPK3 (p-RIPK3) and actin. The latter used as a protein loading control. Ctr: BMDMs treated with TNF, SMAC mimetics and a caspase-8 inhibitor. n(WT mock) = 2; n(WT infected) = 3; n(*C8/R3*$^{-/-}$ mock) = 2; n(*C8/R3*$^{-/-}$ infected) = 2; n(*R3*$^{-/-}$ mock) = 2; n(*R3*$^{-/-}$ infected) = 2 mice. Each lane corresponds to a biological replicate (lung homogenate of one animal). Blots are representative of 2 independent experiments. **(b-c)** 12-week-old WT and *R3*$^{-/-}$ mice were infected intranasally with 10^4 TCID50 of SARS-CoV-2 strain and monitored for **(b)** percentages of animals that became severely ill (reaching predetermined ethical endpoint) and **(c)** weight loss compared to their initial weight at different dpi (Mean ± SD are shown; n(WT) = 6; n(*R3*$^{-/-}$) = 7 mice). **(d)** Levels of cytokines and chemokines measured by ELISA of lung homogenates of WT and knockout animals 3 days post SARS-CoV-2 infection (n(WT) = 7; n(*C8/R3*$^{-/-}$) = 6; n(*R3*$^{-/-}$) = 9 mice; p-values can be found in the source data file). **(e)** Representative images of immunohistochemistry (IHC) stained lungs for MPO (neutrophils) and CD3 (T cells) of infected WT and gene targeted animals (images are representative of at least 3 animals per genotype). **(f-h)** WT mice were infected with SARS-CoV-2 and treated with the broad-spectrum caspase inhibitor emricasan or vehicle as illustrated in the schematic shown in **(f)** and monitored for **(g)** lung viral burden by TCID50 assay and **(h)** percent weight change compared to initial weight (n = 8 mice per condition). **(i-k)** WT mice were infected and treated with the caspase-8 preferential inhibitor Z-IETD-FMK **(i)** and monitored for **(j)** lung viral burden by TCID50 assay and **(k)** percent weight change compared to initial weight (n(Vehicle) = 9; n(IETD) = 10 mice). Data are presented as mean ± SD unless noted otherwise. Log-rank Mantel-Cox test (b), Wilcoxon rank-sum (d), unpaired two-tailed Student's t test after log10 transformation (g,j) and unpaired two-tailed Student's t test (h,k), statistical tests were performed; *$P < 0.05$ and **$P < 0.01$. f and I were partially Created in BioRender. M. Bader, S. (2025) https://BioRender.com/se0ia8f. Source data are provided as a Source Data file.

Figure S2

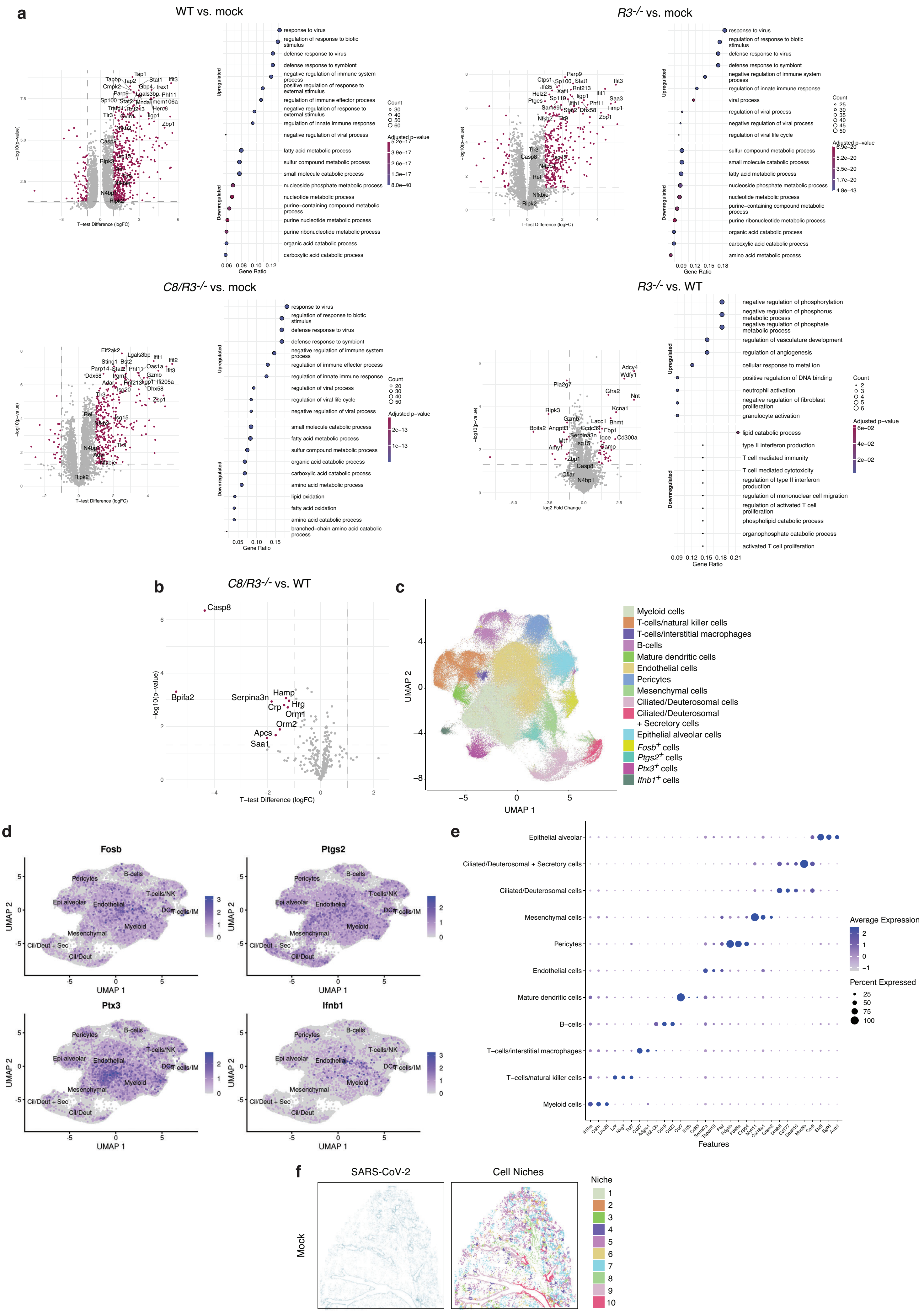
a
WT vs. mock
R3-/- vs. mock
C8/R3-/- vs. mock
R3-/- vs. WT
Upregulated
Downregulated
T−test Difference (logFC)
−log10(p−value)
log2 Fold Change
Gene Ratio
Count
Adjusted p−value
response to virus
regulation of response to biotic stimulus
defense response to virus
defense response to symbiont
negative regulation of immune system process
positive regulation of response to external stimulus
regulation of immune effector process
negative regulation of response to external stimulus
regulation of innate immune response
negative regulation of viral process
viral process
regulation of viral process
regulation of viral life cycle
fatty acid metabolic process
sulfur compound metabolic process
small molecule catabolic process
nucleoside phosphate metabolic process
nucleotide metabolic process
purine−containing compound metabolic process
purine nucleotide metabolic process
purine ribonucleotide metabolic process
organic acid catabolic process
carboxylic acid catabolic process
amino acid metabolic process
lipid oxidation
fatty acid oxidation
amino acid catabolic process
branched−chain amino acid catabolic process
negative regulation of phosphorylation
negative regulation of phosphorus metabolic process
negative regulation of phosphate metabolic process
regulation of vasculature development
regulation of angiogenesis
cellular response to metal ion
positive regulation of DNA binding
neutrophil activation
negative regulation of fibroblast proliferation
granulocyte activation
lipid catabolic process
type II interferon production
T cell mediated immunity
T cell mediated cytotoxicity
regulation of type II interferon production
regulation of mononuclear cell migration
regulation of activated T cell proliferation
phospholipid catabolic process
organophosphate catabolic process
activated T cell proliferation
b
C8/R3-/- vs. WT
Casp8
Bpifa2
Serpina3n
Hamp
Crp
Hrg
Orm1
Orm2
Apcs
Saa1
T−test Difference (logFC)
−log10(p−value)
c
UMAP 1
UMAP 2
Myeloid cells
T-cells/natural killer cells
T-cells/interstitial macrophages
B-cells
Mature dendritic cells
Endothelial cells
Pericytes
Mesenchymal cells
Ciliated/Deuterosomal cells
Ciliated/Deuterosomal + Secretory cells
Epithelial alveolar cells
Fosb+ cells
Ptgs2+ cells
Ptx3+ cells
Ifnb1+ cells
d
Fosb
Ptgs2
Ptx3
Ifnb1
Pericytes
B-cells
T-cells/NK
Epi alveolar
Endothelial
DCs
T-cells/IM
Myeloid
Mesenchymal
Cil/Deut + Sec
Cil/Deut
UMAP 1
UMAP 2
e
Epithelial alveolar
Ciliated/Deuterosomal + Secretory cells
Ciliated/Deuterosomal cells
Mesenchymal cells
Pericytes
Endothelial cells
Mature dendritic cells
B−cells
T−cells/interstitial macrophages
T−cells/natural killer cells
Myeloid cells
Features
Average Expression
Percent Expressed
f
SARS-CoV-2
Cell Niches
Mock
Niche

Fig. S2: Proteomics and spatial transcriptomic analyses of lungs of *C8/R3$^{-/-}$* animals reveal the importance of caspase-8 in the immune response to SARS-CoV-2 infection. (a-b) C57BL/6 (WT), *caspase-8/Ripk3* knockout (*C8/R3$^{-/-}$*) and *Ripk3* knockout (*R3$^{-/-}$*) mice were inoculated intranasally with either mock (media only) or SARS-CoV-2 P21 (10^4 TCID50) and lungs were taken at 3 dpi for bulk proteomics analysis (n(WT mock) = 4; n(WT infected) = 4; n(*C8/R3$^{-/-}$* mock) = 4; n(*C8/R3$^{-/-}$* infected) = 4; (*R3$^{-/-}$* mock) = 4; n(*R3$^{-/-}$* infected) = 4). **(a)** Volcano plots of proteins regulated in mouse lungs at 3 dpi in the diverse comparisons is shown on the left-hand side. Purple: significantly differentially expressed proteins. On the right-hand side, dot plots showing the top 10 enriched GO terms among significantly upregulated (top panel) and downregulated (bottom panel) proteins is shown (all annotations were made using the respective gene names). Pathways were selected based on gene ratio and adjusted p-value. Each dot represents a GO term, with dot size indicating the number of proteins (as gene names) contributing to the term and colour scale representing the adjusted p-value (Benjamini-Hochberg correction). **(b)** Volcano plot of WT vs. *C8/R3$^{-/-}$* comparison showing only the 289 proteins common to the comparisons of SARS-CoV-2 infected WT mice vs. WT mice and SARS-CoV-2 infected *R3$^{-/-}$* mice vs. mock *R3$^{-/-}$* mice, which are not present in the comparison between SARS-CoV-2 infected *C8/R3$^{-/-}$* mice vs. *C8/R3$^{-/-}$* mice. **(c-f)** WT, *C8/R3$^{-/-}$* and *R3$^{-/-}$* mice were inoculated intranasally with either mock (media only) or SARS-CoV-2 and lungs were taken at 3 dpi for spatial transcriptomic analysis using MERSCOPE. **(c)** Unsupervised clustering of transcriptomic profiles revealed distinct cell populations, visualized using Uniform Manifold Approximation and Projection (UMAP). Cells were annotated based on transcriptional similarity to LungMAP reference signatures and grouped into 15 clusters. Each point represents a single cell, coloured by cluster identity as indicated in the legend. **(d)** UMAP plots with the expression (log-transformed counts per cell) of *Fosb, Ifnb1, Ptgs2, Ptx3* genes displayed. Unsupervised clusters were obtained after the removal of transcriptomic counts of the four displayed genes, showing that expression of the displayed genes is not specific to any of the 11 known cell type clusters. **(e)** Dot plot showing the expression levels of canonical marker genes used to define cell clusters following Harmony-based integration of spatial transcriptomic data. Rows represent annotated clusters and columns denote selected marker genes. Dot size reflects the proportion of cells within a cluster expressing the gene, while colour indicates scaled average expression. **(f)** Proportions of cell types within ten spatially-defined “niches”, based on 30 nearest neighbours were defined. Niche distribution in lungs of mock (media only) animals. Left side: spatial maps of SARS-CoV-2-positive (red) and non-infected (grey) regions. Red points mark cells located within 10 µm of an infected region. Right side: Niche maps showing spatial distribution of each of the ten niches across genotypes.

Figure S3

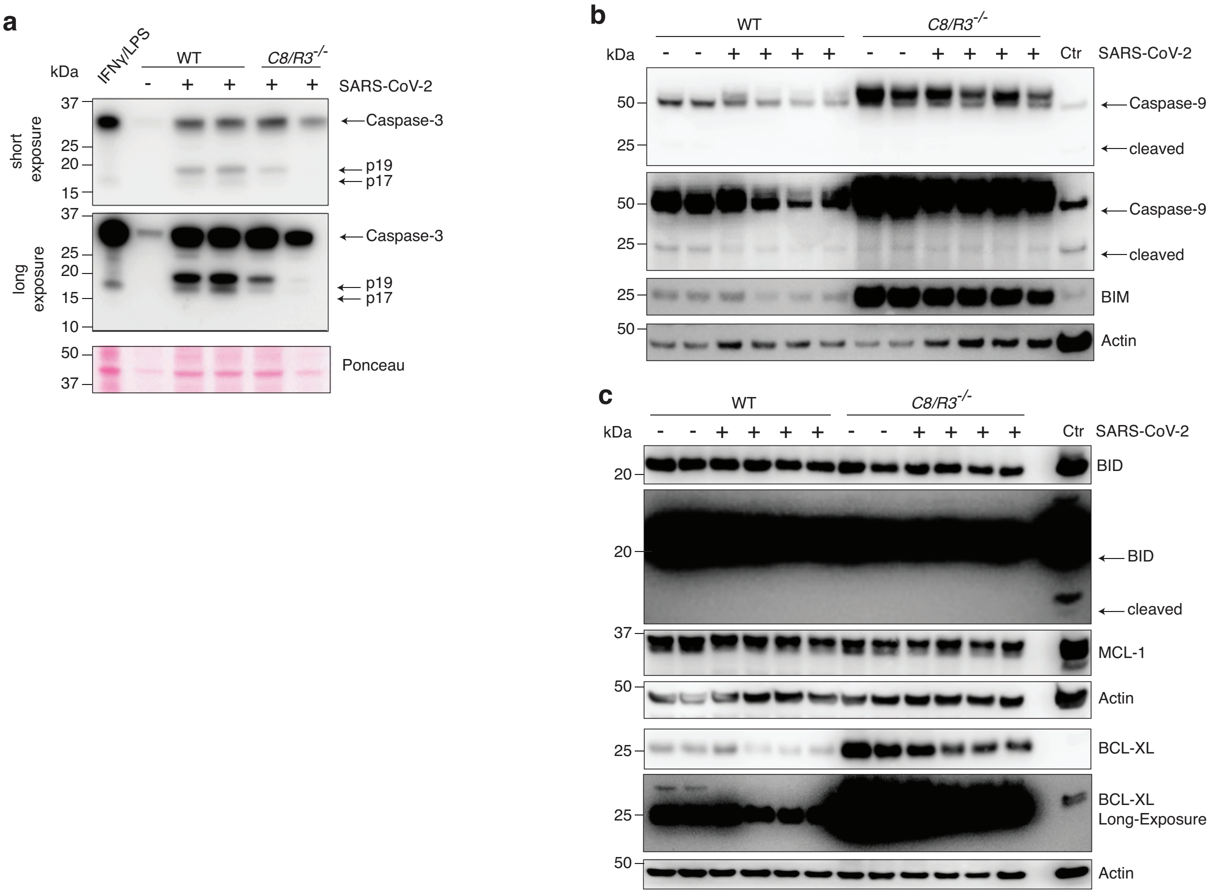
a
IFNγ/LPS
WT
C8/R3-/-
kDa
SARS-CoV-2
short exposure
long exposure
Caspase-3
p19
p17
Ponceau
b
WT
C8/R3-/-
kDa
Ctr
SARS-CoV-2
Caspase-9
cleaved
BIM
Actin
c
WT
C8/R3-/-
kDa
Ctr
SARS-CoV-2
BID
cleaved
MCL-1
Actin
BCL-XL
BCL-XL Long-Exposure
Actin

Fig. S3: Caspase-8 driven disease during SARS-CoV-2 infection is not due to overt apoptosis. (a) Western blot analysis of bronchoalveolar lavage (BAL) from mock (media only) (-) or SARS-CoV-2 infected (+) WT and *C8/R3*$^{-/-}$ mice at 3 days after intranasal SARS-CoV-2 infection (10^4 TCID50). Samples were probed for full-length caspase-3 and cleaved (i.e. activated) caspase-3 (n(WT mock) = 1; n(WT infected) = 2; n(*C8/R3*$^{-/-}$ infected) = 2). **(b)** Western blot analysis of whole lungs from mock (-) or SARS-CoV-2 infected (+) WT and *C8/R3*$^{-/-}$ mice at 3 days after intranasal SARS-CoV-2 infection (10^4 TCID50). Samples were probed for full-length and cleaved caspase-9, BIM, full-length and cleaved BID, MCL-1, BCL-XL and actin, the latter used as a loading control (n(WT mock) = 2; n(WT infected) = 4; n(*C8/R3*$^{-/-}$ mock) = 2; n(*C8/R3*$^{-/-}$ infected) = 4). Each lane corresponds to a biological replicate (lung homogenate of one animal). Blots are representative of 2 independent experiments.

Figure S4

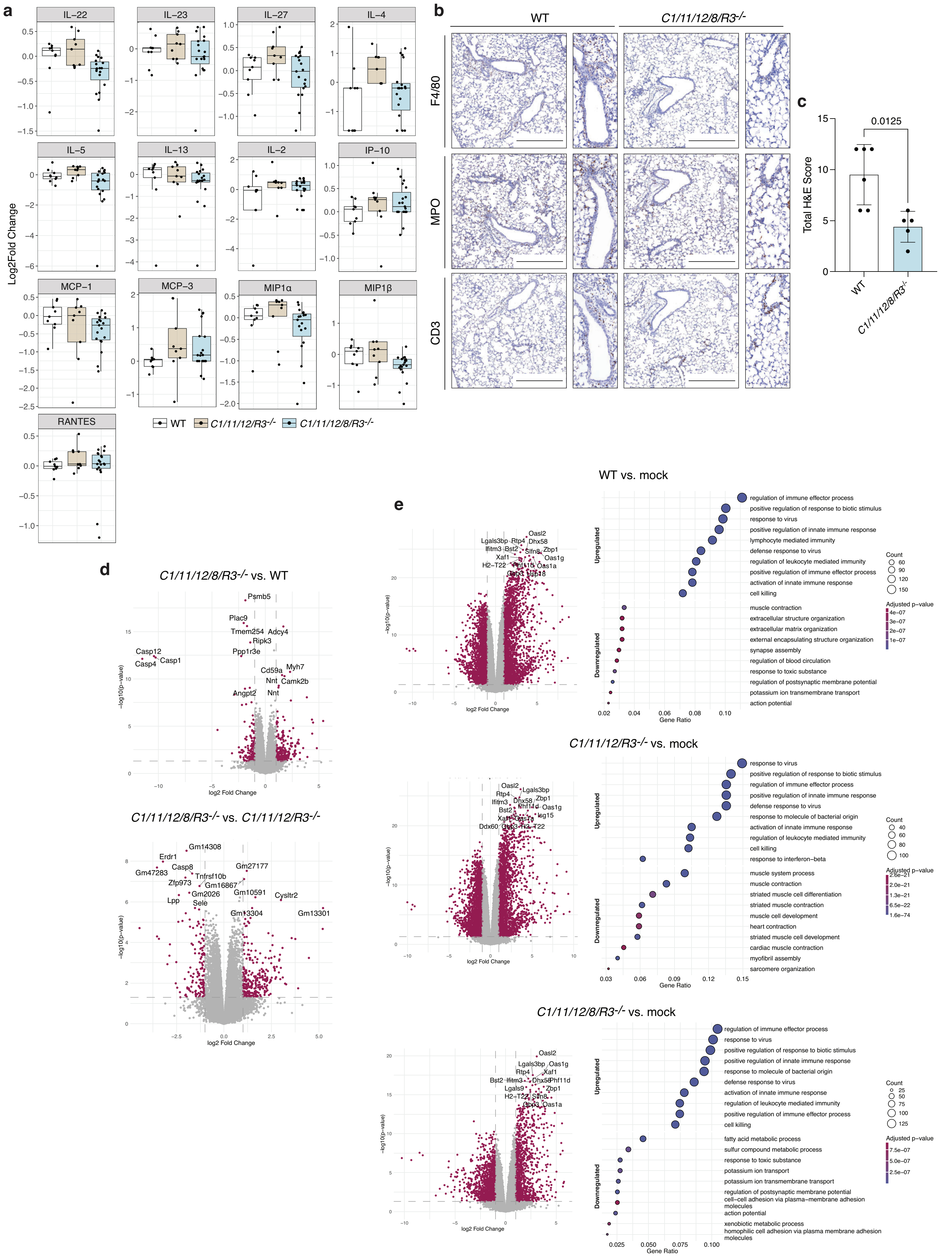
a
IL−22
IL−23
IL−27
IL−4
IL−5
IL−13
IL−2
IP−10
MCP−1
MCP−3
MIP1α
MIP1β
RANTES
Log2Fold Change
WT
C1/11/12/R3-/-
C1/11/12/8/R3-/-
b
WT
C1/11/12/8/R3-/-
F4/80
MPO
CD3
c
Total H&E Score
0.0125
WT
C1/11/12/8/R3-/-
d
C1/11/12/8/R3-/- vs. WT
Psmb5
Plac9
Tmem254
Adcy4
Ripk3
Ppp1r3e
Casp12
Casp4
Casp1
Cd59a
Myh7
Nnt
Camk2b
Angpt2
Nnt
−log10(p−value)
log2 Fold Change
C1/11/12/8/R3-/- vs. C1/11/12/R3-/-
Gm14308
Erdr1
Gm47283
Casp8
Gm27177
Tnfrsf10b
Zfp973
Gm16867
Gm2026
Gm10591
Cysltr2
Lpp
Sele
Gm13304
Gm13301
e
WT vs. mock
Oasl2
Lgals3bp
Rtp4
Dhx58
Ifitm3
Bst2
Slfn8
Zbp1
Xaf1
Oas1g
H2−T22
Phf11d
Oas1a
Gbp3
Usp18
Upregulated
Downregulated
regulation of immune effector process
positive regulation of response to biotic stimulus
response to virus
positive regulation of innate immune response
lymphocyte mediated immunity
defense response to virus
regulation of leukocyte mediated immunity
positive regulation of immune effector process
activation of innate immune response
cell killing
muscle contraction
extracellular structure organization
extracellular matrix organization
external encapsulating structure organization
synapse assembly
regulation of blood circulation
response to toxic substance
regulation of postsynaptic membrane potential
potassium ion transmembrane transport
action potential
Gene Ratio
Count
Adjusted p−value
C1/11/12/R3-/- vs. mock
Oasl2
Lgals3bp
Rtp4
Zbp1
Ifitm3
Dhx58
Phf11d
Oas1g
Bst2
Isg15
Xaf1
Oas1a
Ddx60
Gbp3
H2−T22
response to virus
positive regulation of response to biotic stimulus
regulation of immune effector process
positive regulation of innate immune response
defense response to virus
response to molecule of bacterial origin
activation of innate immune response
regulation of leukocyte mediated immunity
cell killing
response to interferon−beta
muscle system process
muscle contraction
striated muscle cell differentiation
striated muscle contraction
muscle cell development
heart contraction
striated muscle cell development
cardiac muscle contraction
myofibril assembly
sarcomere organization
C1/11/12/8/R3-/- vs. mock
Oasl2
Lgals3bp
Oas1g
Rtp4
Xaf1
Bst2
Ifitm3
Dhx58
Phf11d
Lgals9
Zbp1
H2−T22
Slfn8
Gbp3
Oas1a
regulation of immune effector process
response to virus
positive regulation of response to biotic stimulus
positive regulation of innate immune response
response to molecule of bacterial origin
defense response to virus
activation of innate immune response
regulation of leukocyte mediated immunity
positive regulation of immune effector process
cell killing
fatty acid metabolic process
sulfur compound metabolic process
response to toxic substance
potassium ion transport
potassium ion transmembrane transport
regulation of postsynaptic membrane potential
cell−cell adhesion via plasma−membrane adhesion molecules
action potential
xenobiotic metabolic process
homophilic cell adhesion via plasma membrane adhesion molecules

Fig. S4: Caspase-8 drives inflammation and viral dissemination independently of Caspases-1/-11/-12 and RIPK3. (a) Levels of cytokines and chemokines measured by ELISA in lung homogenates of mice of the indicated genotypes at 3 days post-infection (dpi) with SARS-CoV-2 (10^4 TCID50) (n(WT) = 9; n(*C1/11/12/8/R3*$^{-/-}$) = 9; n(*C1/11/12/R3*$^{-/-}$) = 19 mice per genotype; p-values can be found in the source data file). *B*oxplots depict the median and interquartile range (IQR). Whiskers extend to the furthest data point within 1.5 times the IQR from each box end. Wilcoxon rank-sum statistical tests were performed). **(b)** WT and the indicated gene knockout mice were infected with SARS-CoV-2 (10^4 TCID50) and lungs were taken for immunohistochemistry analysis using antibodies to detect F4/80 (macrophages), MPO (neutrophils) and CD3 (T cells). Images are representative of at least 3 animals per genotype. **(c)** Histological changes in the lungs of infected mice based on H&E staining from Fig. 5f were graded by a pathologist. The score is based on the percentage of lesions: 0 = normal, 1 < 10%, 2 = 10-25%, 3 = 25-50%, 4 > 50%. The sum of the histological scores for WT and *C1/11/12/8/R3*$^{-/-}$ mice are shown (n(WT) = 6; n(*C1/11/12/8/R3*$^{-/-}$) = 5; unpaired t-test was performed). **(d-e)** WT, *C1/11/12/R3*$^{-/-}$ and *C1/11/12/8/R3*$^{-/-}$ mice were inoculated intranasally with SARS-CoV-2 (10^4 TCID50) or mock (media only) and lungs were taken at 3 dpi for RNAseq analysis (n(WT mock) = 4; n(WT infected) = 6; n(*C1/11/12/R3*$^{-/-}$ mock) = 4; n(*C1/11/12/R3*$^{-/-}$ infected) = 6; n(*C1/11/12/8/R3*$^{-/-}$ mock) = 4; n(*C1/11/12/8/R3*$^{-/-}$ infected) = 6). **(d)** Volcano plot of comparisons of infected *C1/11/12/8/R3*$^{-/-}$ vs. infected WT mice and infected *C1/11/12/8/R3*$^{-/-}$ mice vs. infected *C1/11/12/R3*$^{-/-}$ mice are shown. **(e)** Volcano plots and pathway enrichment analysis of significantly differentially expressed genes identified from SARS-CoV-2 infected WT and gene knockout mice vs. their respective mock controls using Hallmark gene sets. Negative $\log_{10}$ FDR-adjusted P values associated with each pathway are plotted; dot sizes correspond to the proportion of all genes from that pathway that were found to be significantly differentially expressed in each comparison (Gene Ratio). Source data are provided as a Source Data file.

Figure S5

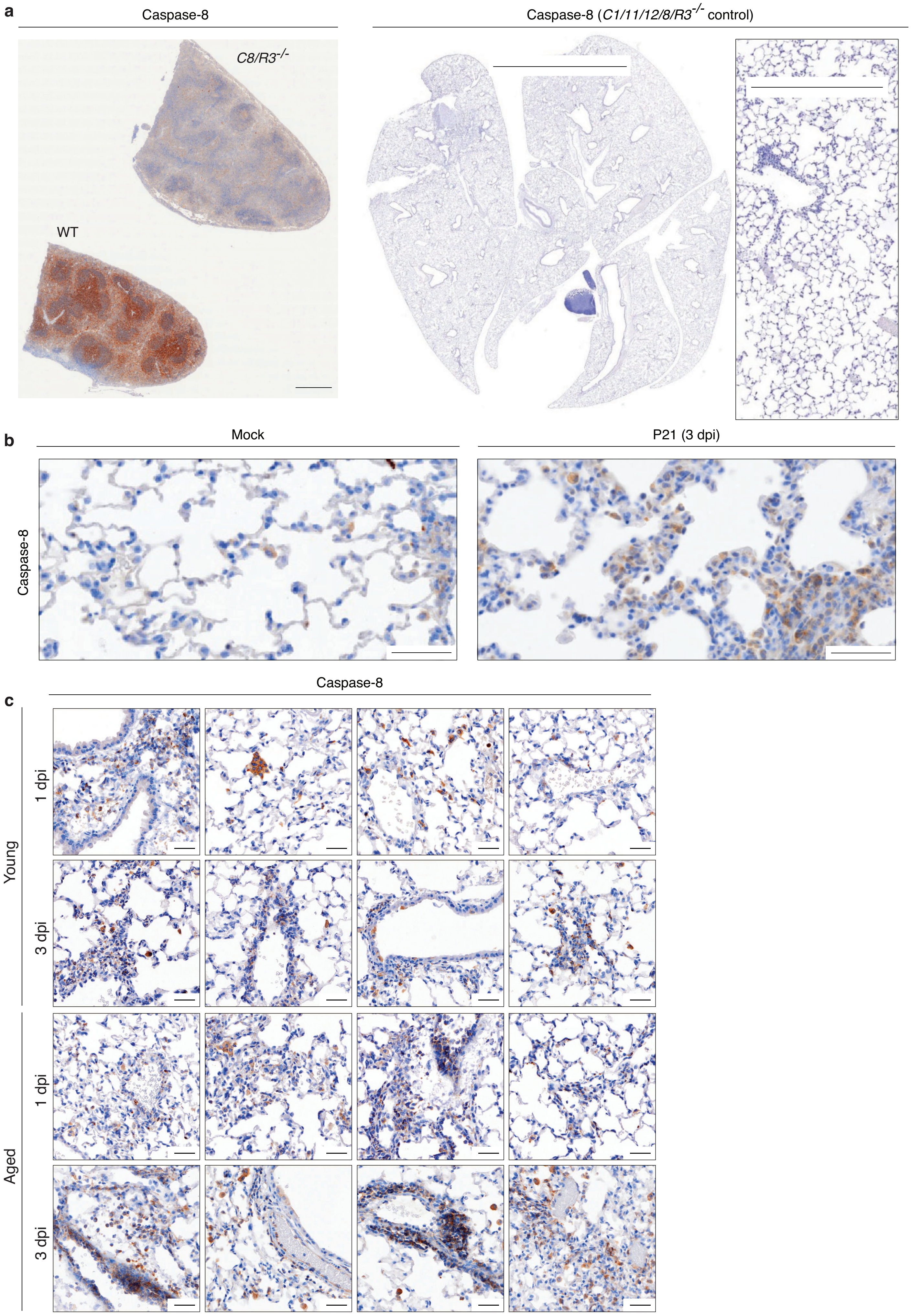
a
Caspase-8
C8/R3-/-
WT
Caspase-8 (C1/11/12/8/R3-/- control)
b
Mock
P21 (3 dpi)
Caspase-8
c
Caspase-8
Young
1 dpi
3 dpi
Aged
1 dpi
3 dpi

Fig. S5*:* Caspase-8 levels are increased in lungs of SARS-CoV-2 infected mice. **(a)** Representative images of tissues stained for caspase-8. Spleens of WT and *C1/11/12/8/R3*$^{-/-}$ animals were used and positive and negative controls of caspase-8 staining respectively (left panel). *C1/11/12/8/R3*$^{-/-}$ mice were infected intranasally with 10^4 TCID50 of SARS-CoV-2 strain and lungs were collected at 3 days post-infection (dpi) for histological analysis as a negative control for caspase-8 staining upon infection (right panel)*.* **(b)** Representative images of lungs from mock (inoculated with media only) and SARS-CoV-2 infected animals (10^4 TCID50) of indicated genotypes at 3 dpi that had been IHC stained for caspase-8. Scale bar = 50 µm **(c)** Representative images of lungs from SARS-CoV-2 infected young (7 weeks) and aged (6 months) WT animals. IHC staining for caspase-8 was performed. All images are representative of at least 3 animals per genotype and condition. Scale bar = 50 µm.

Supplementary Table 1

Table S1: Mouse strains used in this study.

Strain	Description	Source	Reference
C57BL/6	These mice were imported from Jackson Laboratories and were originally designated as C57BL/6J. JAX do not allow the use of the J suffix for strains which have been maintained away from Jax for more than 5-10 generations.	Jackson Laboratories and WEHI	Strain #000664
C57BL/6N	Wild-type C57BL/6N	Charles River	n/a
Ripk3$^{-/-}$	Whole body *Ripk3* deletion	Vishva Dixit (Genentech)	[1]
Casp8/Ripk3$^{-/-}$	Whole body *Caspase-8* and *Ripk3* double deletion. *Casp8*$^{+/-}$ were originally made and gifted to the Pellegrini Lab by SM Hedrick (UC) [2]. Heterozygous animals were then crossed to *Ripk3*$^{-/-}$ [1] to generate *Casp8*$^{-/-}$ *Ripk3*$^{-/-}$.	SM Hedrick (UC) and Pellegrini Lab (WEHI)*	[1,2]
C1/11/12/R3$^{-/-}$	Whole body *Caspase-1, Caspase-11, Caspase-12 and Ripk3* quadruple deletion. Were obtained by crossing of animals from *Casp8/Ripk3*$^{-/-}$ strain with *C1/11/12*$^{-/-}$at WEHI by Marco Herold.	Taconic Artemis GmbH (Cologne, Germany) and Herold Lab (WEHI)	and [1,2]
C1/11/12/8/R3$^{-/-}$	Whole body *Caspase-1, Caspase-11, Caspase-12, Caspase-8 and Ripk3* quintuple deletion. Were obtained by crossing of animals from *Casp8/Ripk3*$^{-/-}$ strain with *C1/11/12*$^{-/-}$at WEHI by Marco Herold.	Taconic Artemis GmbH (Cologne, Germany) and Herold Lab (WEHI)	Taconic Artemis GmbH and [1,2]
Ifn-γ$^{-/-}$	Whole body *Ifn-γ* deletion	Jackson Laboratories (#002287)	[3]
Tnf/Ifn-γ$^{-/-}$	Whole body *Tnf* and *Ifn-γ* double deletion was made by crossing *Tnf* [4] *and Ifn-γ*$^{/-}$ [3] strains.	Johanna Groom (WEHI)	[3,4]
Il-6$^{-/-}$	Whole body *Il-6* deletion	Ian Wicks (WEHI)	[5]
p50$^{-/-}$	Whole body *p50NF-kB1 deletion (NFKB1)*	WEHI	[6]

Uncropped Western Blots (Supplementary Figures)

Supplementary Figure 1a

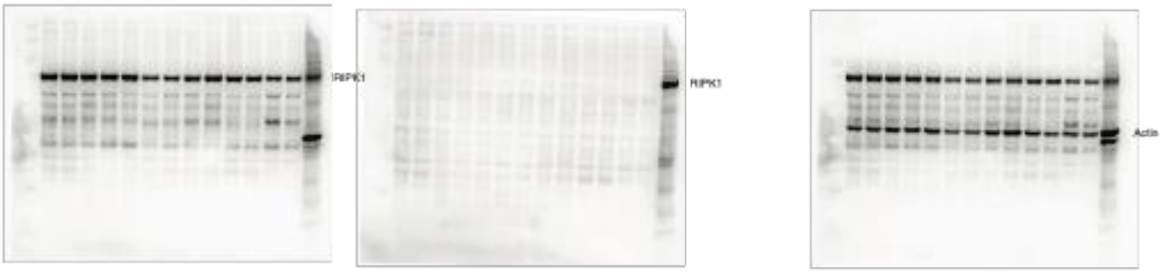

Supplementary Figure 3a

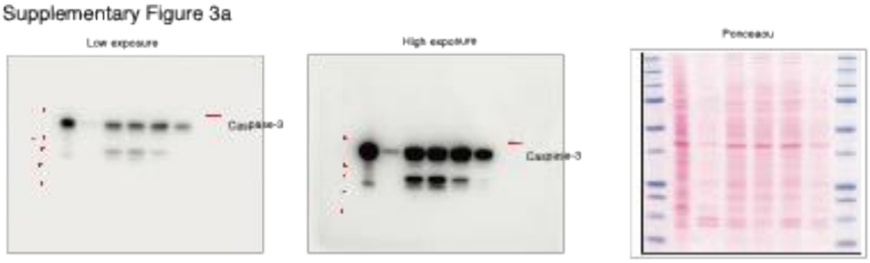

Supplementary Figure 3b

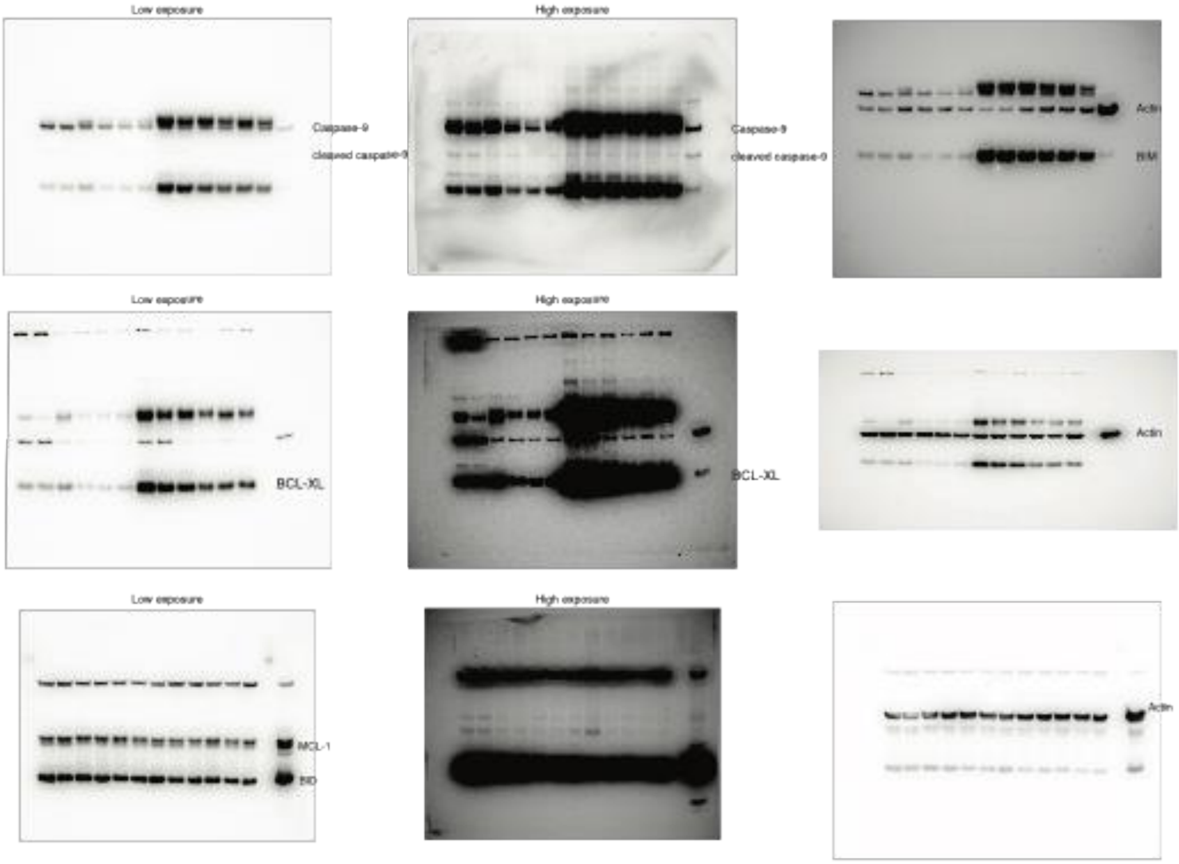

Supplementary References

1 Newton, K., Sun, X. & Dixit, V. M. Kinase RIP3 is dispensable for normal NF-kappa Bs, signaling by the B-cell and T-cell receptors, tumor necrosis factor receptor 1, and Toll-like receptors 2 and 4. *Mol Cell Biol* **24**, 1464-1469 (2004). https://doi.org/10.1128/mcb.24.4.1464-1469.2004

2 Beisner, D. R., Chu, I. H., Arechiga, A. F., Hedrick, S. M. & Walsh, C. M. The requirements for Fas-associated death domain signaling in mature T cell activation and survival. *J. Immunol.* **171**, 247-256 (2003). https://doi.org/10.4049/jimmunol.171.1.247

3 Dalton, D. K. *et al.* Multiple defects of immune cell function in mice with disrupted interferon-gamma genes. *Science* **259**, 1739-1742 (1993). https://doi.org/10.1126/science.8456300

4 Körner, H. *et al.* Distinct roles for lymphotoxin-alpha and tumor necrosis factor in organogenesis and spatial organization of lymphoid tissue. *Eur. J. Immunol.* **27**, 2600-2609 (1997). https://doi.org/10.1002/eji.1830271020

5 Kopf, M. *et al.* Impaired immune and acute-phase responses in interleukin-6-deficient mice. *Nature* **368**, 339-342 (1994). https://doi.org/10.1038/368339a0

6 Sha, W. C., Liou, H. C., Tuomanen, E. I. & Baltimore, D. Targeted disruption of the p50 subunit of NF-kappa B leads to multifocal defects in immune responses. *Cell* **80**, 321-330 (1995). https://doi.org/10.1016/0092-8674(95)90415-8
