# Supplementary material for: Non-apoptotic caspase-8 is critical for orchestrating exaggerated inflammation during severe SARS-CoV-2 infection
Source: Nat Commun. 2025 Nov 13;16:9822. doi: 10.1038/s41467-025-65098-z (PMC12615602; doi:10.1038/s41467-025-65098-z)
Supplement: Supplementary file 2 — Reporting summary [file 41467_2025_65098_MOESM2_ESM.pdf]

nature portfolio

Corresponding author(s): Marcel Doerflinger, Marc Pellegrini, Stefanie M. Bader

Last updated by author(s): Sep 23, 2025

Reporting Summary

Nature Portfolio wishes to improve the reproducibility of the work that we publish. This form provides structure for consistency and transparency in reporting. For further information on Nature Portfolio policies, see our Editorial Policies and the Editorial Policy Checklist.

Statistics

For all statistical analyses, confirm that the following items are present in the figure legend, table legend, main text, or Methods section.

n/a	Confirmed	
☐	☒	The exact sample size (*n*) for each experimental group/condition, given as a discrete number and unit of measurement
☐	☒	A statement on whether measurements were taken from distinct samples or whether the same sample was measured repeatedly
☐	☒	The statistical test(s) used AND whether they are one- or two-sided<br>*Only common tests should be described solely by name; describe more complex techniques in the Methods section.*
☐	☒	A description of all covariates tested
☐	☒	A description of any assumptions or corrections, such as tests of normality and adjustment for multiple comparisons
☐	☒	A full description of the statistical parameters including central tendency (e.g. means) or other basic estimates (e.g. regression coefficient) AND variation (e.g. standard deviation) or associated estimates of uncertainty (e.g. confidence intervals)
☐	☒	For null hypothesis testing, the test statistic (e.g. *F*, *t*, *r*) with confidence intervals, effect sizes, degrees of freedom and *P* value noted<br>*Give P values as exact values whenever suitable.*
☒	☐	For Bayesian analysis, information on the choice of priors and Markov chain Monte Carlo settings
☐	☒	For hierarchical and complex designs, identification of the appropriate level for tests and full reporting of outcomes
☐	☒	Estimates of effect sizes (e.g. Cohen's *d*, Pearson's *r*), indicating how they were calculated

Our web collection on statistics for biologists contains articles on many of the points above.

Software and code

Policy information about availability of computer code

Data collection

The following machines and software were used for data collection:

RNAseq data: Agilent Tapestation; Illumina NextSeq 500.
Histology: VS200 scanner (Olympus).
Cytokines and chemokines: Bio-Plex 200 system (Bio-Rad).
Proteomics: Orbitrap Astral (Thermo Fisher Scientific).
CosMx™ Spatial Molecular Imaging (Nanostring) and CosMx instrument with the Technology Access Program (Nanostrin, Seattle).
MERSCOPE platform.

For further details please see methods section.

Data analysis

The following software and packages were used for data analysis:

Adobe Illustrator v28.6
GraphPad Prism (v10.2.3) GraphPad Software Schneider
RStudio (v1.4.1743-4, R 4.2.0, 4.5.0) - R packages: ggplot2, ggpubr, openxlsx, reshape2, v2.24.0, Rsubread (v4.2), edgeR
MERFISH packages: Seurat 123 (v5.3.0), SCTransform(), IntegrateLayers(method=HarmonyIntegration), (Harmony v1.2.3), FindNeighbors(), FindClus-ters(resolution=0.2), FindAllMarkers()), Lung CellRef atlas, Seurat BuildNicheAssay()
Interactions between proteins were examined using STRING
QuPath (v0.4.3)

Vizgen Post-Processing tool (VPT); MERlin (Vizgen, software version v233).
Western Blots were quantified using Image lab software (v6.1)
Prism v9.3.1

For further details please see methods section.

For manuscripts utilizing custom algorithms or software that are central to the research but not yet described in published literature, software must be made available to editors and reviewers. We strongly encourage code deposition in a community repository (e.g. GitHub). See the Nature Portfolio guidelines for submitting code & software for further information.

Data

Policy information about availability of data

All manuscripts must include a data availability statement. This statement should provide the following information, where applicable:

- Accession codes, unique identifiers, or web links for publicly available datasets
- A description of any restrictions on data availability
- For clinical datasets or third party data, please ensure that the statement adheres to our policy

Data Availability

All data is available within this paper or as Supplementary Information. Source data are provided with this paper.
• The mass spectrometry proteomics data have been deposited to the Prote-omeXchange Consortium via the PRIDE 127 partner repository with the da-taset identifier PXD057656 (https://www.ebi.ac.uk/pride/archive/projects/ PXD057656).
• CosMx data from human patient explants is available in Zenodo (DOI: 10.5281/zenodo.15597679). https://zenodo.org/records/15597680.
• MERFISH data and processed objects used for performing the analysis shown in the figures in the manuscript are available from Zenodo (DOI: 10.5281/ zenodo.15719666).
• The RNA-seq data generated in this study have been deposited at GEO (GSE282408) and are publicly available as of the date of publication.

Code Availability
• Codes for Merscope processing and Cellpose cell-segmentation are availa-ble from Zenodo (DOI: 10.5281/zenodo.15719804).

Research involving human participants, their data, or biological material

Policy information about studies with human participants or human data. See also policy information about sex, gender (identity/presentation), and sexual orientation and race, ethnicity and racism.

Reporting on sex and gender	Donor 1 (CosMx) was a 63-year-old female and donor 2 (Histology) was a 58-year-old female.
Reporting on race, ethnicity, or other socially relevant groupings	Donor 1 (CosMx) was a never smoker who contributed healthy lung tissue, collected after lobectomy of the right lower lobe. Donor 2 (Histology) was a current smoker who contributed healthy lung tissue, collected during lobectomy of the left lower lobe. N/A
Population characteristics	N/A
Recruitment	Written informed consent was obtained from all patients by the Victorian Cancer Biobank prior to inclusion in the study.
Ethics oversight	WEHI Human Research Ethics Committee (HREC, 10/04LR).

Note that full information on the approval of the study protocol must also be provided in the manuscript.

Field-specific reporting

Please select the one below that is the best fit for your research. If you are not sure, read the appropriate sections before making your selection.

☒ Life sciences ☐ Behavioural & social sciences ☐ Ecological, evolutionary & environmental sciences

For a reference copy of the document with all sections, see nature.com/documents/nr-reporting-summary-flat.pdf

Life sciences study design

All studies must disclose on these points even when the disclosure is negative.

Sample size	For in vivo experiments, in accordance with the 3Rs, the smallest sample size was chosen that could give a significant difference (less than 0.05 type 1 error probability at 0.8 power). Pilot experiments were used to estimate the sample size such that an appropriate statistical test could yield significant results. The exact n numbers used in each experiment are indicated in the figure legends.<br>Sample sized for experiments involving drug treatments varied depending on compound availability.
Data exclusions	Animals that did not show productive infection (TCID50 under the limit of detection) were excluded in experiments that compared viral loads (TCID50) and weight loss between different viral strains or mouse genotypes.

Replication	All key experiments were reproduced in 2-3 independent biological repeats (n values in figure legends). Where possible, data from independent repeats were pooled; where pooling was not appropriate, a representative experiment or replicate is shown and the number of independent repeats is stated.
Randomization	For mouse studies, mice were randomly allocated to infection groups.
Blinding	Pathologists were blinded during scoring of histological images and TCID50.

Reporting for specific materials, systems and methods

We require information from authors about some types of materials, experimental systems and methods used in many studies. Here, indicate whether each material, system or method listed is relevant to your study. If you are not sure if a list item applies to your research, read the appropriate section before selecting a response.

Materials & experimental systems

n/a	Involved in the study
☐	☒ Antibodies
☐	☒ Eukaryotic cell lines
☒	☐ Palaeontology and archaeology
☐	☒ Animals and other organisms
☒	☐ Clinical data
☒	☐ Dual use research of concern
☒	☐ Plants

Methods

n/a	Involved in the study
☒	☐ ChIP-seq
☒	☐ Flow cytometry
☒	☐ MRI-based neuroimaging

Antibodies

Antibodies used

Antibodies used for histology (manufacturer, cat number):
CD3 (Agilent A045201), MPO (Agilent A039829), F4/80 (WEHI in-house antibody) or SARS-CoV-2 nucleocapsid (abcam ab271180).

Antibodies used for western blots:
caspase-3 (9662, Cell Signaling Technology), cFLIP (1:1000, D5J1E, Cell Signaling Technology), caspase-8 (3B10, in-house), cleaved (i.e. activated) caspase-8 (D5B2, Cell Signaling Technology) and N4BP1 (Abcam ab133610), HRP-conjugated goat secondary antibodies (anti-rabbit IgG, anti-rat IgG) (Southern Biotech, Birmingham, AL, USA)

Validation

All antibodies listed in the previous section were validated by the manufacturer and/or by previous studies.

Information on the validation of antibodies for flow cytometry can be found as stated below:

Abcam: https://go.myabcam.com/BiophysicalQuality#:~:text=That's%20why%20we're%20continually,400%20added%20last%20year%20alone.
The high quality of our antibodies is founded on a range of precise validation techniques. Biophysical testing builds on these tools to let you know our antibodies in detail, so you can have confidence in your results no matter what kind of assay set-up you're using. They include: Recombinant technology, extensive application testing, advanced validation, knock-out validation.

Agilent: https://www.agilent.com/en/product/immunohistochemistry
Our immunohistochemistry products, including our IHC instruments, provide high staining quality, improved standardization and increased efficiency enabling the ultimate goal of increased patient safety. The broad portfolio of Agilent Dako products is continuously enhanced with new generations of carefully selected and clinically relevant reagents and instruments.

Cell Signaling Technology: https://www.cellsignal.com/about-us/cst-antibody-validation-principles?srsltid=AfmBOooRDsp_5yPOeZfGzBEe9-VJNl6rrB42Sm0t2kZE-hGMZHtXEO6T
At CST, we validate all of the antibodies we sell in-house using rigorous, application-specific testing. And we make 95% of the antibodies we sell. Having this kind of control over antigen design, antibody development, validation, and production is the best way we know to ensure that our products will consistently help you achieve reliable, reproducible results in your lab. And it's why we can guarantee that CST® antibodies will perform as expected, every time.

Southern Biotech: https://www.southernbiotech.com/#:~:text=About%20SouthernBiotech%20*%20In%2DHouse%20Production%20&%20Validation.,Flexible%2C%20Customizeable%20Services.%20*%20Responsive%20Customer%20Support.

WEHI: In house- antibodies are validated through the WEHI histology facility. Different conditions of staining (buffers, dilutions, incubation conditions) are tested in initial optimization runs, including internal controls (tissue positive and negative) to establish antibody specificity and staining protocol.

Eukaryotic cell lines

Policy information about cell lines and Sex and Gender in Research

Cell line source(s)	Vero cells: kidney epithelial cells from African green monkey.
Authentication	Vero cells were purchased from ATCC (clone CCL-81) and used to make a cell bank. Vials are thawed and the cells were used until passage 30.
Mycoplasma contamination	All cell lines tested negative for mycoplasma contamination by PCR.
Commonly misidentified lines (See ICLAC register)	No commonly misidentified cell lines were used in the study.

Animals and other research organisms

Policy information about studies involving animals; ARRIVE guidelines recommended for reporting animal research, and Sex and Gender in Research

Laboratory animals	All mice except for Ripk3-/- mice were on a C57BL/6J background. Ripk3-/- mice were on a C57BL/6N background and experiments were performed comparing Ripk3-/- to WT C57BL/6N mice. 6-8-week-old, 11-week-old or 6-8-month-old animals were used and aged-matched within experiments. For more details, please refer to Methods sections of the manuscript.
Wild animals	No wild animals were used.
Reporting on sex	Both sexes were used, as direct comparison between SARS-CoV-2 P21 infected male and female C57BL/6 mice did not show any differences in viral loads (TCID50) or weight loss.
Field-collected samples	No field-collected samples were used.
Ethics oversight	All procedures and mouse strains were reviewed and approved by The Walter and Eliza Hall Institute of Medical Research Animal Ethics Committee and were conducted in accordance with the Prevention of Cruelty to Animals Act (1986) and the Australian National Health and Medical Research Council Code of Practice for the Care and Use of Animals for Scientific Purposes (1997).

Note that full information on the approval of the study protocol must also be provided in the manuscript.

Plants

Seed stocks	N/A
Novel plant genotypes	N/A
Authentication	N/A
